# BOA-Derived Volumetric CT Body Composition Provides Prognostic Information Beyond BMI in Surgically Treated Head and Neck Squamous Cell Carcinoma: A Retrospective Cohort Study

**DOI:** 10.3390/cancers18152371

**Published:** 2026-07-23

**Authors:** Charlotte Kamrath, Markus Blaurock, Nadine Hoepfner, Philipp Dittmann, Matthis Ebel, Fabian Paperlein, Chia-Jung Busch, Mark Oliver Wielpütz, Verena Wagner

**Affiliations:** 1 Department of Otorhinolaryngology, Head and Neck Surgery, Universitätsmedizin Greifswald, 17475 Greifswald, Germany; 2 Department of Maxillofacial and Plastic Surgery, Universitätsmedizin Greifswald, 17475 Greifswald, Germany; 3 Department of Diagnostic Radiology and Neuroradiology, Universitätsmedizin Greifswald, 17475 Greifswald, Germanymark.wielpuetz@med.uni-greifswald.de (M.O.W.)

**Keywords:** body composition, sarcopenia, head and neck cancer, BOA algorithm, computed tomography, volumetric analysis, prognosis, cervical vertebra

## Abstract

**Background/Objectives:** To evaluate whether automated three-dimensional volumetric body composition metrics from the Body and Organ Analysis (BOA) algorithm provide prognostic information beyond body mass index (BMI) for overall survival (OS) in surgically treated head and neck squamous cell carcinoma (HNSCC), and to compare cervical (C1–C7) measurements with thoracic and abdominal regional comparators. **Methods:** This single-center retrospective cohort study included 391 surgically treated HNSCC patients (2010–2019). Pretreatment CT scans were processed using the open-source BOA algorithm to obtain volumetric muscle, bone, and adipose measurements across cervical (C1–C7), thoracic, and abdominal regions. Composite indices—Sarcopenia Index (SI = Muscle/Bone) and Myosteatotic Fat Index (MFI = IMAT/TAT)—were derived; Cerv SI denotes the volumetric C1–C7 ratio. The predefined primary Cox model adjusted for age, sex, tumor site, UICC stage, treatment, HN-CCI, and secondary malignancy. **Results:** Of 391 patients (91% male, median follow-up 5.0 years, 141 deaths), BMI was not independently associated with OS (HR 0.88, *p* = 0.15). Cerv SI was associated with OS in the minimally adjusted model (HR 0.71, 95% CI 0.58–0.88, *p* = 0.002) and remained independent in the primary clinically adjusted model (HR 0.72, 95% CI 0.58–0.90, *p* = 0.004; C-index 0.67). When BMI and Cerv SI were entered together, Cerv SI remained independently associated (HR 0.72, *p* = 0.006) while BMI was null (HR 1.01, *p* = 0.95). **Conclusions:** BOA-derived cervical volumetric body composition is associated with overall survival beyond BMI and is obtainable from routine head and neck CT. Prospective validation and comparison with established two-dimensional C3/L3 methods are warranted before clinical use.

## Simple Summary

Head and neck cancer is one of the most common cancers worldwide, and patients’ outcomes vary widely even when the tumor stage is similar. One reason is that body composition—how much muscle, bone, and fat a person has—affects how well someone tolerates surgery and recovery. Body mass index is the usual way to measure this, but it cannot tell muscle from fat. In this study, we used a new computer algorithm that automatically measures muscle, bone, and fat from routine neck CT scans. In 391 patients who had surgery for head and neck cancer, the ratio of neck muscle to bone was independently associated with survival, while body mass index was not. Because most head and neck cancer patients already have neck CT scans for staging, this approach could give clinicians an objective additional risk marker without additional image acquisition or burden to the patient.

## 1. Introduction

Head and neck squamous cell carcinoma (HNSCC) represents one of the most common malignancies worldwide [1], with treatment decisions guided primarily by tumor staging, site, and comorbidity burden [2]. Radical treatment encompasses surgery, definitive radiotherapy and chemoradiation, and, in selected sites and stages, organ-preserving approaches, including interventional radiotherapy (brachytherapy), with systemic therapy used in the definitive, adjuvant, and recurrent or metastatic settings; the present cohort addresses the surgically treated population specifically. Despite advances in surgical techniques and adjuvant therapy, individual prognosis varies substantially, highlighting the need for additional prognostic biomarkers that capture patient-level vulnerability beyond tumor characteristics.

Sarcopenia—the pathological loss of skeletal muscle mass and function—has emerged as a robust independent prognostic factor across multiple malignancies. In HNSCC specifically, a recent multi-level meta-analysis of 63 studies (n = 14,804) confirmed that pretreatment radiologically defined sarcopenia is consistently associated with worse survival (log OR 0.81, *p* < 0.001) and increased treatment complications [3]. Earlier meta-analyses by Hua et al. [4] (HR 1.97, 95% CI 1.71–2.26 for OS) and Wong et al. [5] (HR 1.98, 95% CI 1.64–2.39) corroborated these findings across heterogeneous HNSCC populations, as did a further meta-analysis by Koh et al. [6], which reported a consistent association between sarcopenia and mortality in head and neck cancer.

Traditionally, image-based sarcopenia in HNSCC has been assessed using single-slice cross-sectional muscle area at the third lumbar (L3) or third cervical (C3) vertebral level, measured either manually or semi-automatically. This approach, while validated, has limitations: it captures a two-dimensional snapshot of three-dimensional tissue, is subject to inter-observer variability, and requires specific vertebral landmarks that may not be included in routine head and neck imaging. Meanwhile, body mass index (BMI), the most widely used anthropometric measure, fails to distinguish between muscle and fat mass. This gives rise to the “BMI paradox” in HNSCC, whereby overweight patients paradoxically show improved survival compared to normal-weight patients [7].

Recent advances in deep learning have enabled fully automated, three-dimensional volumetric body composition analysis (BCA) from routine clinical CT scans, as summarized in a systematic review of automated 3D segmentation methods [8]. The Body and Organ Analysis (BOA) algorithm [9], developed at the University Hospital Essen, combines body-composition segmentation with the TotalSegmentator framework to enable automated tissue quantification throughout the entire scanned volume. BOA segments muscle, bone, and multiple adipose tissue compartments (subcutaneous, visceral, intramuscular, epicardial, pericardial) with high accuracy (Dice >0.95) and processes a complete CT in under one minute [9,10,11]. Importantly, BOA provides volumetric measurements—not just single-slice areas—potentially capturing body composition more completely.

Several groups have demonstrated that volumetric BCA may carry additional prognostic value compared to single-slice analysis. In lung cancer, Künnemann et al. [12] showed that a volumetric Sarcopenia Index (Muscle/Bone ratio) outperformed conventional L3-based single-slice measurements in predicting OS. Jung et al. [13] demonstrated in 36,317 UK Biobank participants that volumetric body composition measures predicted mortality independently of BMI, with associations attenuated when using single-slice metrics. However, in a colorectal cancer cohort, Anyene et al. [14] found that single-slice L3 and volumetric metrics had similar prognostic performance, suggesting the advantage may be context-dependent.

For HNSCC patients, in whom abdominal imaging is frequently unavailable, the possibility of deriving prognostic body-composition information from routine neck CT is of particular clinical relevance. Cervical muscle measurements at C3 have been validated against L3 references [15,16,17,18], and recent work by Barajas Ordonez et al. [19] (n = 904) confirmed that both skeletal muscle area and muscle radiation attenuation at C3 predict OS in HNSCC. However, these studies used single-slice 2D measurements. Whether volumetric 3D cervical body composition from automated algorithms provides comparable or superior prognostic value to thoracic and abdominal measurements has not been investigated.

The aim of this study was threefold: (1) to evaluate whether BOA-derived 3D volumetric body composition indices provide prognostic information beyond BMI for overall survival (OS) and disease-free survival (DFS) in surgically treated HNSCC; (2) to assess whether cervical C1–C7 volumetric measurements provide prognostic information comparable to thoracic and abdominal measurements; and (3) to evaluate the robustness of the primary finding after adjustment for established oncologic confounders including tumor stage.

## 2. Materials and Methods

### 2.1. Study Design and Participants

This retrospective single-center cohort study included consecutive patients with histologically confirmed HNSCC who underwent primary curative surgery ± adjuvant (chemo)radiation at the Department of Otorhinolaryngology, Head and Neck Surgery, Universitätsmedizin Greifswald between 2010 and 2019. Patients were eligible if they had (a) a documented first diagnosis date, (b) at least one pretreatment CT scan obtained before surgery and encompassing the neck and, where available, the thorax and abdomen, and (c) available follow-up data. The study was reported in accordance with the Strengthening the Reporting of Observational Studies in Epidemiology (STROBE) guidelines for cohort studies; because the prognostic marker is derived by an automated deep-learning imaging pipeline, we additionally drew on applicable items from the TRIPOD+AI [20] and CLAIM [21] reporting frameworks for artificial-intelligence and medical-imaging studies. Before initiation, the ethics committee approved the study with the registry number BB 099/24 on 5 July 2024. Patients who received definitive (chemo)radiation without surgery or non-standard adjuvant chemotherapy alone (n = 13) were excluded because the study was designed as a surgically treated cohort; these cases had been captured in the source database but fell outside the intended surgical inclusion criteria, so that the cohort uniformly reflected surgically treated HNSCC.

### 2.2. Body Composition Analysis

Pretreatment CT scans were processed using the open-source BOA algorithm (v0.1.3; github.com/UMEssen/Body-and-Organ-Analysis accessed on 20 July 2026) [9]. BOA employs a multiresolution nnU-Net architecture to segment body regions and tissues and combines it with TotalSegmentator [11] for organ identification. The algorithm provides volumetric measurements (in mL) for the following tissue compartments within defined body regions: skeletal muscle, bone, subcutaneous adipose tissue (SAT), visceral adipose tissue (VAT), intramuscular/intermuscular adipose tissue (IMAT), epicardial adipose tissue (EAT), pericardial adipose tissue (PAT), and total adipose tissue (TAT). Measurements were obtained separately for three body regions: the abdominal cavity (excluding extremities), the thoracic cavity (excluding extremities), and the cervical spine (C1–C7, excluding extremities). Quality control was performed using the BOA status output; only measurements with “OK” status were retained.

CT examinations were acquired as part of routine clinical staging. For the primary analysis, the staging CT closest to surgery was used; across the cohort, the staging CT was obtained a median of 17 days before surgery (IQR 10–25 days), with 86% acquired within 30 days before surgery and all scans (100%) obtained before the operative date. Where multiple eligible scans were available, the scan with the most complete cervical/thoracic/abdominal coverage and an acceptable BOA quality status was selected. CT examinations were acquired on a range of scanners in routine clinical use over the 2010–2019 study period. Across all processed CT series, the large majority were obtained on Siemens systems—most frequently the SOMATOM Sensation 16 and SOMATOM Definition Flash—with smaller numbers acquired on Philips and General Electric scanners. Reconstructed slice thickness ranged from 0.625 to 5 mm (median 2 mm). Reconstruction kernel, intravenous contrast phase, and tube voltage were not uniformly retrievable across this retrospective cohort (see Limitations).

In addition to the BOA internal “OK” status filter, a visual quality-control review was performed in a random subset of cervical segmentations and in outlier cases. Segmentations were checked for gross vertebral-level mismatch, incomplete C1–C7 coverage, dental-artifact-related segmentation failure, and erroneous inclusion or exclusion of non-target tissue. Three investigators independently reviewed a random subset of 35 cervical segmentations. No significant segmentation artifacts or errors were identified; specifically, no cases of dental-artifact-related segmentation failure, vertebral-level mismatch, or incomplete C1–C7 coverage were observed, and no cases required exclusion or re-processing on this basis. Representative cervical segmentations from this review are shown in Figure S2.

### 2.3. Derived Composite Indices

Based on prior work in lung cancer [12], two composite indices were calculated for each body region: (1) Sarcopenia Index (SI) = Muscle volume/Bone volume, a measure of relative muscle mass normalized to skeletal frame size; and (2) Myosteatotic Fat Index (MFI) = IMAT volume/TAT volume, a measure of fatty infiltration of muscle relative to total adiposity, reflecting muscle quality. For cervical analysis, tissue volumes from C1 through C7 were summed into a single cervical composite measurement. Two cervical metrics are distinguished throughout and are defined here. Cerv SI (cervical Sarcopenia Index) denotes the BOA-derived volumetric Muscle/Bone ratio computed across the summed C1–C7 tissue volumes. C3 SI (single-vertebra Sarcopenia Index) denotes the BOA-derived volumetric Muscle/Bone ratio restricted to the tissue volume assigned to the C3 vertebral level alone. Both are volumetric measurements extracted from the same three-dimensional segmentation, and these terms are used consistently in the text and tables. Neither is equivalent to the conventional C3 Sarcopenia Index used in most HNSCC studies, which is a two-dimensional cross-sectional skeletal muscle area (cm^2^) traced manually or semi-automatically on a single axial slice at the mid-body of C3 and typically normalized to height^2^, or converted to an estimated L3-equivalent value using published prediction formulas. The BOA-derived C3 SI therefore differs from the conventional 2D C3 index in dimensionality (volume versus area), normalization (bone volume versus height^2^), segmentation method (fully automated versus manual or semi-automatic), and the anatomical extent of tissue sampled. Because the conventional 2D C3 measurement was not available in this cohort, comparisons between the C3 SI and the Cerv SI should be interpreted within the BOA-derived feature space and not as a test of the conventional method.

### 2.4. Clinical Data and Outcomes

Demographic and clinical variables were extracted from institutional databases: age at diagnosis, sex, BMI (kg/m^2^), tumor site, TNM staging (UICC 7th and 8th editions, reflecting the editions in use across the 2010–2019 inclusion period), treatment modality, Head and Neck Charlson Comorbidity Index (HN-CCI), ASA physical status classification, preoperative leukocyte count, maximum postoperative C-reactive protein (CRP), secondary malignancy status (“secondary malignancy” as documented in clinical records), p16 status, and Clavien–Dindo graded complications and infections. ASA classification was used as a functional status measure because ECOG performance status data were incompletely and inconsistently documented in these retrospective records. This is a recognized limitation of retrospective HNSCC cohorts, as ECOG assessment is inherently subjective and subject to considerable inter-rater variability [22,23], and ASA has been shown to outperform ECOG as a predictor of overall survival in HNSCC patients treated with adjuvant (chemo)radiation [24]. The primary outcome was overall survival (OS), defined as time from first diagnosis to death from any cause or last follow-up. The secondary outcome was disease-free survival (DFS), defined as the time from diagnosis to the first documented recurrence or death from any cause, whichever occurred first. UICC stage was modeled as an ordinal stage-group variable (I–IV) as documented in the clinical record; this approach harmonized stage-group information across the UICC editions in use during the inclusion period.

### 2.5. Statistical Analysis

Continuous predictors were standardized (z-scored) for Cox regression to enable comparison of hazard ratios (HR) across variables with different scales. To address multiple testing, we predefined a hierarchy of analyses: the primary imaging marker was the cervical C1–C7 SI; secondary markers were the thoracic SI, thoracic MFI, and abdominal SI; exploratory markers were all remaining single-compartment volumes and the single-vertebra C3 comparator. In a sensitivity analysis, treating UICC stage as a categorical rather than ordinal covariate did not materially change the association between cervical SI and overall survival (HR 0.73, *p* = 0.006).

Univariate Cox proportional hazards models were fitted for each predictor. The primary clinically adjusted model (reported in Table 4) included the cervical SI together with age, sex, tumor site (larynx, oral cavity, oropharynx, and hypopharynx, with larynx as the reference category), UICC stage, treatment modality (surgery alone, surgery plus chemoradiation, and surgery plus radiotherapy, with surgery alone as the reference category), HN-CCI, and secondary malignancy; a parallel specification replaced UICC stage with T and N category separately. A minimally adjusted model including only HN-CCI and secondary malignancy (reported in Table 3) was retained as a supportive analysis. Treatment modality was included to account for broad differences in treatment course, but treatment coefficients were not interpreted causally because treatment selection reflects tumor risk, patient fitness, and multidisciplinary decision-making; the cervical SI association was essentially unchanged in models with and without treatment (HR 0.75 without versus HR 0.72 with treatment). Because maximum postoperative CRP is measured after the imaging biomarker is acquired and may lie on the causal pathway between frailty, surgical morbidity, and survival, leukocytes and CRP were not included in the primary pretreatment model but were examined in a separate sensitivity model (Table 3, Model E). Sensitivity analyses included stratification by imaging availability. The proportional hazards assumption was assessed using Schoenfeld residuals; in the primary clinically adjusted model, the cervical SI satisfied the proportional hazards assumption (*p* = 0.45), with minor deviations noted for sex and UICC stage that did not affect the cervical SI estimate; the global test was significant (χ^2^ = 20.80, df = 11, *p* = 0.036), driven by these adjustment covariates rather than by the cervical SI (Supplementary Table S3); the scaled Schoenfeld residuals for the cervical SI are shown in (Supplementary Figure S3). To confirm that the significant global test did not distort the exposure estimate, the primary model was refitted, stratifying the baseline hazard separately on each covariate showing a departure from proportionality (UICC stage, sex, and tumor site). The cervical SI hazard ratio was essentially unchanged in each case: stratified by UICC stage (HR 0.74, 95% CI 0.59–0.93, *p* = 0.011), by sex (HR 0.72, 95% CI 0.57–0.90, *p* = 0.004), and by tumor site (HR 0.72, 95% CI 0.57–0.90, *p* = 0.004), confirming that the proportional-hazards departures among the adjustment covariates do not materially affect the cervical SI estimate. Simultaneous stratification on all three covariates was not pursued as the primary sensitivity analysis because the resulting fragmentation of risk sets in this sample size markedly reduces precision. The linearity of BMI was assessed by comparing a restricted cubic spline model (four knots) with a linear term using a likelihood-ratio test, which showed no evidence of non-linearity (χ^2^ = 0.67, df = 3, *p* = 0.88); BMI was therefore modeled as a linear term. Log-rank tests with median-split dichotomization were used for Kaplan–Meier survival curves. Harrell’s concordance index (C-index) was computed for discriminative ability. Pearson correlation coefficients quantified inter-regional agreement. Statistical significance was set at *p* < 0.05 (two-sided). Analyses were performed using Python 3.12 with the lifelines and scipy packages.

**Figure 1:**
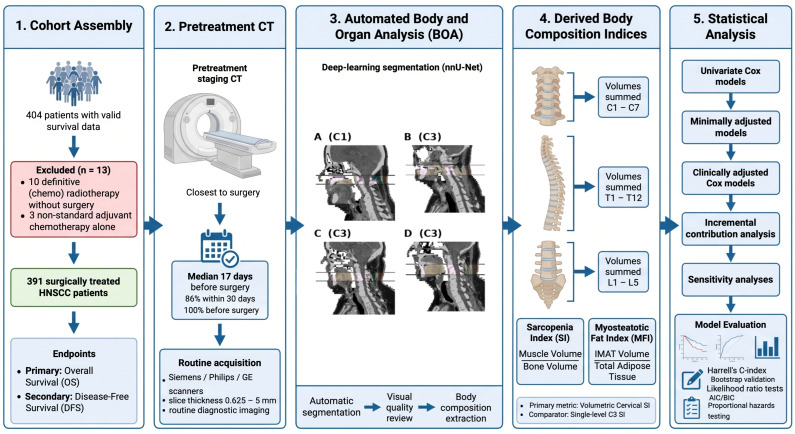
Visual framework of the study. Schematic overview of the analytical pipeline: cohort assembly and exclusion criteria; acquisition of routine pretreatment staging CT; automated body composition segmentation using the BOA algorithm across the cervical (C1–C7), thoracic, and abdominal regions; derivation of the composite indices (Sarcopenia Index = Muscle/Bone; Myosteatotic Fat Index = IMAT/TAT) and of the single-vertebra C3 comparator; and the predefined hierarchy of survival analyses, from univariate models through the minimally adjusted supportive model to the primary clinically adjusted model, with overall survival as the primary and disease-free survival as the secondary endpoint. Created in BioRender. Blaurock, M. (2026). https://BioRender.com/oaugmfs.

The overall analytical workflow of the study is summarized in Figure 1.

## 3. Results

### 3.1. Cohort Characteristics

Of 404 patients in the database with valid survival data, 391 surgically treated HNSCC patients were included in the final analysis after exclusion of 13 patients with definitive nonsurgical treatment or non-standard adjuvant chemotherapy alone, using an administrative censoring cutoff of 28 January 2025 for patients confirmed alive (median follow-up: 5.0 years; IQR: 2.6–6.3). There were 356 males (91%) and 35 females (9%), with a mean age of 60.9 years. p16 status was tested in 34 patients (8.7%), of whom 17 (50%) were p16-positive. TNM (UICC 7th and 8th editions) staging was retrievable for all 391 patients (100%). The mean BMI was 26.6 ± 5.3 kg/m^2^. Treatment approaches included surgery alone (n = 173, 44%), surgery with radiation (n = 84, 21%), and surgery with chemoradiation (n = 134, 34%). A total of 141 deaths (36.1%) were recorded. Secondary malignancy was documented in 67 cases (17.1%). BOA-processed CT data were available for 256 patients (cervical, C1–C7), 222 (thoracic), and 159 (abdominal). Single-level C3 segmentation data—used as an in-sample BOA-derived single-vertebra comparator—were available for 248 patients. The overview is displayed in Table 1. Imaging availability was not random and was associated with UICC stage (χ^2^ = 70.29, df = 12, *p* < 0.001; Supplementary Table S1): patients with multi-region imaging had a higher proportion of UICC III–IV disease than patients with cervical-only imaging, so regional comparisons are interpreted as exploratory. The cohort flow and per-region BOA availability are summarized in Supplementary Figure S1.

### 3.2. Univariate Survival Analysis

In univariate Cox regression with standardized predictors (Table 2), BMI showed no significant association with OS (HR 0.84, *p* = 0.052, C-index 0.55). Among individual tissue volumes, thoracic muscle (HR 0.85, *p* = 0.22) and thoracic SAT (HR 0.79, *p* = 0.05) demonstrated non-significant protective trends. The composite indices performed significantly better: thoracic SI (*p* = 0.002 by log-rank; C-index 0.602), cervical SI (*p* = 0.002, C-index 0.604), and thoracic MFI (*p* < 0.001; C-index 0.621) all showed significant discrimination. Median OS for patients with thoracic SI above the median was 8.9 years, versus 4.2 years for those below the median.

Notably, raw cervical muscle volume alone was not prognostic (*p* = 0.47), whereas the cervical SI (Muscle/Bone ratio) was strongly associated with OS (*p* < 0.001), supporting frame-size normalization using bone volume. In sex-stratified analysis, thoracic SI and cervical SI were significant in males (n = 356), but the female subgroup (n = 35) was underpowered. ASA classification was a significant univariate predictor of OS (HR 1.55 per SD, 95% CI 1.30–1.85, *p* < 0.001). Patients with ASA 3–4 had a median OS of 6.3 years versus 11.9 years for ASA 1–2 (log-rank *p* < 0.001), with mortality rates of 44.1% and 29.0%, respectively. The BOA-derived single-vertebra C3 Sarcopenia Index was also non-prognostic (HR 1.01, 95% CI 0.83–1.24, *p* = 0.899), with a C-index of 0.520 indicating no discriminative ability over chance (Table 2).

### 3.3. Multivariate Survival Analysis

Table 3 shows the results of the minimally adjusted multivariate Cox regression. In models adjusted for HN-CCI and secondary malignancy:

- Cervical SI was independently associated with longer OS (HR 0.71, 95% CI 0.58–0.88, *p* = 0.002)
- Thoracic MFI was associated with worse OS (HR 1.35, 95% CI 1.11–1.63, *p* = 0.002)
- Thoracic SI was independently associated with OS (HR 0.72, 95% CI 0.57–0.90, *p* = 0.005)
- BMI remained non-significant (HR 0.88, *p* = 0.15)

Secondary malignancy was a strong independent confounder across all models (HR 1.31–1.49, *p* ≤ 0.002). HN-CCI was borderline significant (HR 1.15–1.20, *p* = 0.04–0.16). In a sensitivity model that additionally included preoperative leukocytes and maximum postoperative CRP (Table 3, Model E)—both measured after the imaging biomarker and therefore at risk of mediator bias—the cervical SI remained associated with OS (HR 0.70, 95% CI 0.54–0.91, *p* = 0.008). Neither leukocytes nor maximum postoperative CRP contributed independently in this model (both *p* > 0.7).

Minimally adjusted models are presented first for comparability with prior body-composition studies; the predefined primary clinically adjusted models are presented subsequently in Table 4.

**Table 3:** Minimally adjusted and sensitivity Cox proportional hazards models for overall survival. HRs are per 1-SD increase. These models adjust for HN-CCI and secondary malignancy (with leukocytes and CRP in the sensitivity model, Model E) and are retained as supportive analyses; the primary clinically adjusted models are shown in Table 4.

Model/Variable	n	HR	95% CI	*p*-Value
Model A: Thor SI + HN-CCI + Secondary Malignancy (n = 221, 85 events)				
**Thor** **Sarc. Index**	221	0.716	0.57–0.90	**0.005**
HN-CCI		1.145	0.92–1.42	0.225
**Secondary malignancy**		**1.382**	1.15–1.66	**<0.001**
Model B: Thor MFI + HN-CCI + Secondary Malignancy (n = 221, 85 events)				
**Thor MFI (IMAT/TAT)**	221	**1.345**	1.11–1.63	**0.002**
HN-CCI		1.109	0.89–1.38	0.353
**Secondary malignancy**		**1.382**	1.15–1.66	**<0.001**
Model C: Cerv SI + HN-CCI + Secondary Malignancy (n = 254, 102 events)				
**Cerv Sarc. Index**	254	**0.714**	0.58–0.88	**0.002**
HN-CCI		1.109	0.91–1.35	0.305
**Secondary malignancy**		**1.313**	1.11–1.55	**0.002**
Model D: BMI + HN-CCI + Secondary Malignancy (n = 376, 133 events)				
BMI	376	0.876	0.73–1.05	0.154
**HN-CCI**		**1.233**	1.04–1.46	**0.016**
**Secondary malignancy**		**1.488**	1.29–1.71	**<0.001**
Model E: Cerv SI + HN-CCI + Secondary Malignancy + Leuko + CRP (sensitivity) (n = 148, 63 events)				
**Cerv Sarc. Index**	148	**0.701**	0.54–0.91	**0.008**
HN-CCI		**1.202**	0.94–1.54	0.149
**Secondary malignancy**		**1.296**	1.04–1.61	**0.021**
Baseline leukocytes		0.566	0.00–968.82	0.881
Max CRP postop		0.770	0.15–3.92	0.753
Model F: Cerv SI + ASA + HN-CCI + Secondary Malignancy (n = 254, 102 events)				
**Cerv Sarc. Index**	254	**0.757**	0.61–0.94	**0.011**
HN-CCI		1.023	0.83–1.26	0.833
**Secondary malignancy**		**1.317**	1.11–1.56	**0.001**
**ASA**		1.256	1.00–1.57	**0.046**
Model G: BMI + ASA + HN-CCI + Secondary Malignancy (n = 376, 133 events)				
BMI	376	0.839	0.70–1.00	0.055
HN-CCI		1.076	0.90–1.29	0.424
**Secondary malignancy**		**1.446**	1.25–1.67	**<0.001**
**ASA**		**1.506**	1.24–1.83	**<0.001**
Model H: C3 SI + HN-CCI + Secondary Malignancy (n = 246, 99 events)				
C3 Sarc. Index	246	0.966	0.79–1.19	0.742
HN-CCI		1.167	0.96–1.42	0.126
**Secondary malignancy**		**1.364**	1.15–1.61	**<0.001**
Model I: C3 SI + Cerv SI + HN-CCI + Secondary Malignancy (n = 246, 99 events)				
C3 Sarc. Index (single-vertebra)	246	0.991	0.81–1.21	0.933
**Cerv Sarc. Index**		**0.722**	0.58–0.89	**0.002**
HN-CCI		1.134	0.93–1.38	0.216
**Secondary malignancy**		**1.285**	1.08–1.53	**0.004**

Model E is a postoperative inflammatory marker sensitivity model that includes baseline leukocytes and maximum postoperative CRP. Models F and G assess the incremental prognostic value of ASA. In Model F, both cervical SI and ASA remain associated with OS, suggesting that they provide partially complementary prognostic information. In Model G, ASA remains strongly associated with OS, whereas BMI shows only a weak association. Models H and I compare the BOA-derived single-vertebra C3 SI with the volumetric C1–C7 SI: the C3 SI is not prognostic, while the C1–C7 SI remains independently associated with OS. HR < 1 indicates a protective effect. Bold indicates *p* < 0.05.

**Table 4:** Primary clinically adjusted Cox proportional hazards models for overall survival, adjusted for established oncologic confounders (age, sex, tumor site, UICC stage, treatment modality, HN-CCI, and secondary malignancy). HRs are per 1-SD increase for continuous predictors. Reference categories: tumor site = Larynx; treatment modality = Surgery alone. Bold indicates *p* < 0.05. For each model the number of events, Harrell’s concordance index (C), the Akaike and Bayesian information criteria (AIC = −2·log L + 2k; BIC = −2·log L + k·log(E), where k is the number of model parameters and E the number of events), and the likelihood-ratio test (LRT) of the full model against the intercept-only model on the same sample are given in the model header row. Treatment coefficients are not interpreted causally (see Statistical analysis). Model X8 additionally enters BMI alongside the cervical SI in the same adjusted model to assess incremental value directly.

Model/Variable	n	HR	95% CI	*p*-Value
Model X1: Cerv SI + age + sex + site + UICC + tx + HN-CCI + 2nd malignancy (n = 254, 102 events; C = 0.67; AIC = 969.6; BIC = 998.5; LRT χ ^2^ = 37.9, df = 11, *p* < 0.001)				
**Cervical Sarcopenia Index**	**254**	**0.721**	**0.58–0.90**	**0.004**
Age		1.105	0.87–1.40	0.411
Sex (male)		1.099	0.85–1.41	0.465
HN-CCI		1.096	0.89–1.35	0.392
**Secondary malignancy**		**1.328**	**1.12–1.58**	**0.001**
UICC stage		1.033	0.80–1.34	0.806
Site: Hypopharynx		0.941	0.48–1.84	0.860
Site: Oral cavity		0.680	0.39–1.17	0.166
Site: Oropharynx		0.759	0.41–1.40	0.379
Treatment: Surgery + CRT		1.993	1.08–3.67	0.027
Treatment: Surgery + RT		1.356	0.76–2.42	0.304
Model X2: Cerv SI + age + sex + site + T + N + tx + HN-CCI + 2nd malignancy (n = 254, 102 events; C = 0.70; AIC = 963.9; BIC = 995.4; LRT χ ^2^ = 45.6, df = 12, *p* < 0.001)				
**Cervical Sarcopenia Index**	254	**0.766**	**0.61–0.96**	**0.022**
Age		1.138	0.90–1.44	0.289
Sex (male)		1.102	0.86–1.42	0.451
HN-CCI		1.094	0.88–1.35	0.407
**Secondary malignancy**		**1.305**	**1.09–1.56**	**0.003**
**T category**		**1.353**	**1.09–1.68**	**0.006**
N category		1.049	0.81–1.36	0.723
Site: Hypopharynx		0.872	0.45–1.70	0.688
Site: Oral cavity		0.762	0.44–1.33	0.337
Site: Oropharynx		0.947	0.50–1.79	0.867
Treatment: Surgery + CRT		1.585	0.86–2.93	0.143
Treatment: Surgery + RT		1.033	0.59–1.82	0.909
Model X3: Cerv SI + ASA + age + sex + site + UICC + tx + HN-CCI + 2nd malignancy (n = 254, 102 events; C = 0.68; AIC = 968.4; BIC = 999.9; LRT χ ^2^ = 41.0, df = 12, *p* < 0.001)				
**Cervical Sarcopenia Index**	254	**0.757**	**0.60–0.95**	**0.018**
ASA		1.242	0.98–1.58	0.076
Age		1.055	0.83–1.34	0.661
Sex (male)		1.111	0.86–1.43	0.415
HN-CCI		1.028	0.82–1.28	0.810
**Secondary malignancy**		**1.329**	**1.12–1.58**	**0.001**
UICC stage		1.015	0.78–1.32	0.912
Site: Hypopharynx		0.909	0.46–1.80	0.786
Site: Oral cavity		0.687	0.40–1.19	0.180
Site: Oropharynx		0.836	0.45–1.56	0.573
Treatment: Surgery + CRT		2.025	1.09–3.76	0.025
Treatment: Surgery + RT		1.397	0.78–2.50	0.259
Model X4: Thor SI + age + sex + site + UICC + tx + HN-CCI + 2nd malignancy (n = 221, 85 events; C = 0.68; AIC = 793.0; BIC = 819.9; LRT χ ^2^ = 32.0, df = 11, *p* < 0.001)				
**Thoracic Sarcopenia Index**	221	**0.730**	**0.56–0.95**	**0.017**
Age		1.060	0.83–1.36	0.643
Sex (male)		1.086	0.80–1.48	0.599
HN-CCI		1.196	0.95–1.51	0.129
**Secondary malignancy**		**1.434**	**1.19–1.73**	**<0.001**
UICC stage		1.055	0.79–1.41	0.713
Site: Hypopharynx		0.730	0.36–1.49	0.386
Site: Oral cavity		0.649	0.36–1.17	0.148
Site: Oropharynx		0.850	0.44–1.64	0.628
Treatment: Surgery + CRT		1.874	0.93–3.78	0.079
Treatment: Surgery + RT		1.686	0.87–3.25	0.119
Model X5: Thor MFI + age + sex + site + UICC + tx + HN-CCI + 2nd malignancy (n = 221, 85 events; C = 0.68; AIC = 792.7; BIC = 819.6; LRT χ ^2^ = 32.3, df = 11, *p* < 0.001)				
**Thor MFI (IMAT/TAT)**	221	**1.283**	**1.06–1.55**	**0.011**
Age		1.171	0.92–1.49	0.194
Sex (male)		1.026	0.75–1.40	0.874
HN-CCI		1.148	0.91–1.44	0.236
**Secondary malignancy**		**1.420**	**1.17–1.72**	**<0.001**
UICC stage		1.074	0.80–1.43	0.629
Site: Hypopharynx		0.784	0.38–1.60	0.505
Site: Oral cavity		0.669	0.37–1.21	0.183
Site: Oropharynx		0.895	0.47–1.72	0.739
Treatment: Surgery + CRT		1.722	0.85–3.50	0.133
Treatment: Surgery + RT		1.664	0.86–3.22	0.130
Model X6: BMI + age + sex + site + UICC + tx + HN-CCI + 2nd malignancy (n = 376, 133 events; C = 0.67; AIC = 1371.9; BIC = 1403.7; LRT χ ^2^ = 50.6, df = 11, *p* < 0.001)				
BMI	376	0.907	0.75–1.10	0.317
Age		1.204	0.99–1.46	0.059
Sex (male)		1.015	0.83–1.25	0.886
HN-CCI		1.207	1.01–1.44	0.040
**Secondary malignancy**		**1.510**	**1.30–1.75**	**<0.001**
UICC stage		1.177	0.93–1.49	0.173
Site: Hypopharynx		1.194	0.65–2.19	0.565
Site: Oral cavity		0.832	0.53–1.31	0.425
Site: Oropharynx		1.158	0.68–1.96	0.586
Treatment: Surgery + CRT		1.304	0.76–2.23	0.333
Treatment: Surgery + RT		1.209	0.72–2.03	0.474
Model X8: Cerv SI + BMI + age + sex + site + UICC + tx + HN-CCI + 2nd malignancy (n = 253, 101 events; C = 0.67; AIC = 960.7; BIC = 992.1; LRT χ ^2^ = 37.8, df = 12, *p* < 0.001)				
**Cervical Sarcopenia Index**	**253**	**0.722**	**0.57–0.91**	**0.006**
BMI		1.007	0.81–1.25	0.947
Age		1.110	0.87–1.41	0.395
Sex (male)		1.097	0.85–1.41	0.478
HN-CCI		1.102	0.89–1.36	0.375
**Secondary malignancy**	** **	**1.339**	**1.12–1.60**	**0.001**
UICC stage		1.023	0.79–1.33	0.865
Site: Hypopharynx		0.958	0.48–1.89	0.901
Site: Oral cavity		0.687	0.40–1.19	0.178
Site: Oropharynx		0.728	0.39–1.37	0.324
**Treatment: Surgery + CRT**	** **	**1.982**	**1.07–3.67**	**0.030**
Treatment: Surgery + RT		1.376	0.77–2.46	0.281

### 3.4. ASA Classification: Subanalysis

ASA was a significant univariate predictor (HR 1.55, *p* < 0.001; Table 2). In a multivariate model that included cervical SI, HN-CCI, and secondary malignancy (Table 3, Model F), cervical SI retained its association with OS (HR 0.76, *p* = 0.011). At the same time, ASA remained a contributor (HR 1.26; 95% CI 1.00–1.57; *p* = 0.046). In the extended adjusted model (Table 4, Model X3), the cervical SI remained significant (HR 0.78, *p* = 0.021), whereas ASA was attenuated to borderline significance (HR 1.21, *p* = 0.078). When ASA was included in the BMI-based model (Table 3, Model G), ASA retained strong independent significance (HR 1.51, *p* < 0.001) while BMI was attenuated (*p* = 0.055). This pattern is consistent with cervical SI and ASA capturing partially overlapping yet complementary information about patient vulnerability, whereas BMI does not. ASA correlated moderately with cervical SI (Spearman ρ = –0.36, *p* < 0.001), with HN-CCI (ρ = 0.41, *p* < 0.001), and with age (ρ = 0.24, *p* < 0.001), but not with BMI (ρ = 0.07, *p* = 0.19).

### 3.5. BOA-Derived Single-Vertebra C3 Versus Volumetric C1–C7 Sarcopenia Index

The BOA-derived single-vertebra C3 Sarcopenia Index was also non-prognostic (univariate HR 1.01, 95% CI 0.83–1.24, *p* = 0.899, C-index 0.52) and did not survive multivariate adjustment (Table 3, Model H). The Pearson correlation between the BOA single-vertebra C3 SI and the volumetric C1–C7 SI was near zero (r = 0.04, *p* = 0.55, n = 248). Because the BOA-derived C3 metric is a single-vertebra volumetric measurement, not the conventional manually segmented 2D cross-sectional area used in most HNSCC studies, this comparison is best interpreted within the BOA-derived feature space rather than as a direct refutation of conventional C3-based assessment. In a multivariate model including both metrics (Table 3, Model I), the Cerv SI retained independent significance (HR 0.72, 95% CI 0.58–0.89, *p* = 0.002), whereas the C3 SI contributed no additional information (HR 0.99, *p* = 0.93). The C3 SI showed weak-to-moderate correlations with thoracic SI (r = 0.21, *p* = 0.006) and abdominal SI (r = 0.35, *p* < 0.001), and was more strongly correlated with BMI (r = 0.40, *p* < 0.001) than with the volumetric C1–C7 SI.

### 3.6. Kaplan–Meier Analysis and Discriminative Ability

**Figure 2:**
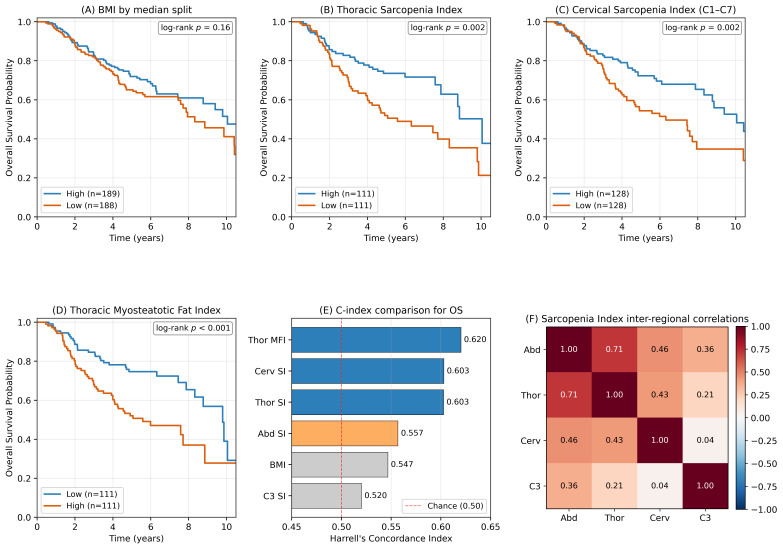
Kaplan–Meier survival curves and concordance indices. (**A**) BMI by median split (*p* = 0.16). (**B**) Thoracic Sarcopenia Index (*p* = 0.002). (**C**) Cervical Sarcopenia Index (Cerv SI; *p* = 0.002). (**D**) Thoracic Myosteatotic Fat Index (*p* < 0.001). (**E**) Harrell’s concordance index (C-index) for overall survival, one univariate model per predictor. Bar colour denotes the discrimination tier (grey, C-index < 0.55, at or near chance; orange, 0.55–0.58; blue, ≥ 0.58); the dashed line at 0.50 marks the C-index expected by chance. (**F**) Pearson correlation matrix of the Sarcopenia Index between body regions.

Figure 2 presents Kaplan–Meier survival curves stratified by median split for the key predictors. Figure 2A shows BMI, for which the curves do not separate (log-rank *p* = 0.16). Figure 2B,C shows the thoracic SI and the Cerv SI, respectively; in both, patients above the median show better survival (*p* = 0.002 for each). Figure 2D shows the thoracic MFI, for which patients above the median show worse survival (*p* < 0.001), consistent with the deleterious direction of its hazard ratio. The C-index comparison (Figure 2E) demonstrates that the body composition metrics (thoracic SI 0.60, thoracic MFI 0.62, Cerv SI 0.60) had modestly higher discrimination than BMI (0.55); the absolute improvements are small. Figure 2F presents the Pearson correlation matrix of the Sarcopenia Index between body regions, showing strong abdominal–thoracic agreement (r = 0.69), moderate cervical–thoracic agreement (r = 0.42), and near-zero correlation between the C3 SI and the Cerv SI (r = 0.04). In paired bootstrap comparisons restricted to patients with both predictors available, Cerv SI showed a modest but significant C-index improvement over BMI (ΔC = +0.087, *p* = 0.038) but did not significantly outperform the C3 SI, ASA classification, or thoracic SI (Supplementary Table S2).

### 3.7. Inter-Regional Correlations

Muscle volumes showed moderate correlations between regions (abdomen–thorax r = 0.32, abdomen–cervical r = 0.25, thorax–cervical r = 0.28). The Sarcopenia Index correlated more strongly between regions: abdomen–thorax r = 0.69 (*p* < 0.001), abdomen–cervical r = 0.47, thorax–cervical r = 0.42. Skeletal Muscle Index (muscle/height^2^) correlated moderately with BMI across regions, confirming that body composition indices capture information distinct from BMI alone. Notably, the single-vertebra C3 SI was uncorrelated with the volumetric C1–C7 SI (r = 0.04, *p* = 0.55), while correlating modestly with BMI (r = 0.40) and with thoracic (r = 0.21) and abdominal (r = 0.35) SI.

### 3.8. Primary Clinically Adjusted Analysis

Because tumor stage, age, sex, primary site, and treatment modality are established prognostic factors in HNSCC, the predefined primary clinically adjusted Cox models included these variables, along with HN-CCI and secondary malignancy, for the four predefined imaging markers; ASA was added in a separate sensitivity variant (Table 4). Surgery alone was used as the reference treatment category and Larynx as the reference tumor site; one patient with a multi-site primary was excluded from the site-adjusted models. Tumor staging was retrieved for all 391 patients; no patients were excluded from extended-adjustment analyses due to missing TNM.

**Figure 3:**
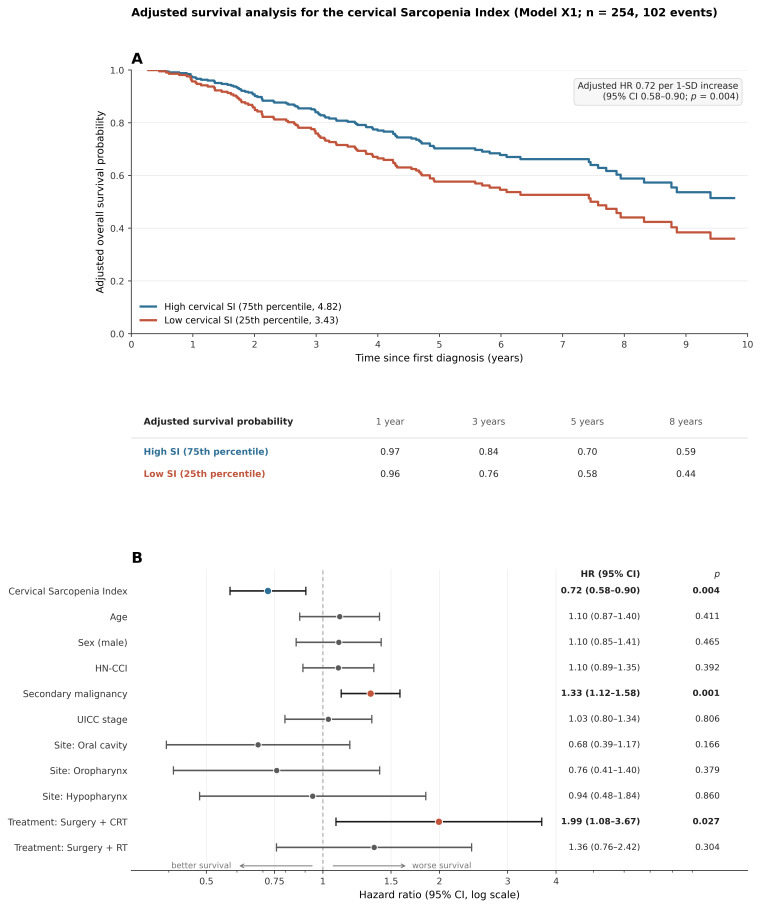
Adjusted survival analysis for the cervical Sarcopenia Index. (**A**) Standardized Cox-adjusted overall survival curves for cervical Sarcopenia Index values at the 25th (3.43) and 75th (4.82) percentiles. Curves were estimated from the primary clinically adjusted Cox model (Model X1; n = 254, 102 events) adjusted for age, sex, tumor site, UICC stage, treatment modality, HN-CCI, and secondary malignancy, and were standardized by marginal averaging of the predicted survival functions over the covariate distribution of the analysis sample. Adjusted survival probabilities at selected time points are shown below the plot. (**B**) Forest plot of the primary clinically adjusted Cox proportional hazards model. Hazard ratios are shown with 95% confidence intervals on a logarithmic scale. HRs for continuous predictors are reported per 1-SD increase. Reference categories: tumor site = Larynx; treatment modality = Surgery alone. Marker colour indicates the direction and statistical significance of each association: blue markers denote statistically significant protective associations (hazard ratio < 1, *p* < 0.05), red markers statistically significant adverse associations (hazard ratio > 1, *p* < 0.05), and grey markers non-significant associations (*p* ≥ 0.05); significant markers are also drawn larger. Hazard ratios and *p*-values shown in bold denote *p* < 0.05.

After extended oncologic adjustment (age, sex, tumor site, UICC stage, treatment modality, HN-CCI, secondary malignancy), the cervical Sarcopenia Index remained independently associated with overall survival (Model X1: HR 0.72, 95% CI 0.58–0.90, *p* = 0.004; n = 254, events = 102; C-index 0.67) (Figure 3). The association persisted when T and N were entered separately rather than as a composite UICC stage (Model X2: HR 0.77, *p* = 0.022). When ASA was added to the extended adjusted model (Model X3), the cervical SI remained significant (HR 0.76, *p* = 0.018) and ASA showed a borderline association (HR 1.24, *p* = 0.076).

Thoracic SI (Model X4: HR 0.73, *p* = 0.017) and thoracic MFI (Model X5: HR 1.28, *p* = 0.011) also retained independent associations under extended adjustment, while BMI (Model X6: HR 0.91, *p* = 0.32) did not. The cervical SI hazard ratio remained near 0.66–0.76 across all model specifications, supporting the robustness of the cervical finding. To test directly whether the cervical SI carries prognostic information beyond BMI, both were entered into the same extended clinically adjusted model on the subset with both measurements available (n = 253, 101 events). The cervical SI remained independently associated with overall survival (HR 0.72, 95% CI 0.57–0.91, *p* = 0.006), whereas BMI was null in the same model (HR 1.01, 95% CI 0.81–1.25, *p* = 0.95). Adding the cervical SI to a model already containing BMI and the clinical covariates significantly improved fit (likelihood-ratio χ^2^ = 7.76, df = 1, *p* = 0.005), whereas adding BMI to a model already containing the cervical SI did not (χ^2^ = 0.00, df = 1, *p* = 0.95) and worsened the AIC. The cervical SI therefore provides prognostic information beyond BMI rather than merely in parallel with it.

To make the incremental contribution of each covariate block explicit, clinical covariates were added sequentially to the cervical SI model on a single fixed complete-case sample (n = 254, 102 events), so that the models are strictly nested (Table 5). The cervical SI hazard ratio was stable across every step (0.66–0.76), confirming that it is not materially influenced by the addition of comorbidity, demographic, oncologic, or treatment covariates. In the reverse ablation, removing the cervical SI from the primary clinically adjusted model significantly worsened model fit (likelihood-ratio χ^2^ = 8.30, df = 1, *p* = 0.004; AIC 975.9 versus 969.6), although its incremental contribution to discrimination once the clinical covariates were present was small (C-index 0.6716 versus 0.6673; ΔC = 0.004). The likelihood-ratio test and the concordance index address different aspects of model performance, and the divergence between them is reported here rather than omitted: the cervical SI adds information to the fitted model, but the absolute gain in discrimination over a model already containing established clinical predictors is modest.

**Table 5:** Stepwise ablation of the primary clinically adjusted model for overall survival. Covariate blocks are added sequentially to the cervical Sarcopenia Index. All steps are fitted on a single fixed complete-case sample (n = 254, 102 events) so that the models are strictly nested, and the likelihood-ratio tests are valid. HRs are per 1-SD increase in the standardized cervical SI. C = Harrell’s concordance index; AIC = Akaike information criterion; BIC = Bayesian information criterion (computed on the number of events); LRT = likelihood-ratio test against the immediately preceding nested model. Step 4 corresponds to Model X1 and Step 5 to Model X3 in Table 4.

Step	n	Ev.	Cerv SI HR (95% CI)	*p*	C	AIC	BIC	LRT vs. Previous
Step 0: Cerv SI alone	254	102	0.661 (0.54–0.81)	<0.001	0.603	971.4	974.0	—
Step 1: + HN-CCI, secondary malignancy	254	102	0.713 (0.58–0.88)	0.002	0.626	965.3	973.1	χ^2^ = 10.09, df = 2, *p* = 0.006
Step 2: + age, sex	254	102	0.730 (0.59–0.91)	0.004	0.631	967.2	980.3	χ^2^ = 2.08, df = 2, *p* = 0.353
Step 3: + tumor site, UICC stage	254	102	0.747 (0.60–0.93)	0.010	0.653	970.4	994.1	χ^2^ = 4.76, df = 4, *p* = 0.313
Step 4: + treatment modality (= Model X1)	254	102	0.721 (0.58–0.90)	0.004	0.672	969.6	998.5	χ^2^ = 4.85, df = 2, *p* = 0.088
Step 5: +ASA (= Model X3)	254	102	0.757 (0.60–0.95)	0.018	0.675	968.4	999.9	χ^2^ = 3.13, df = 1, *p* = 0.077

### 3.9. Disease-Free Survival (Secondary Outcome)

Disease-free survival (DFS) was defined as the time from first diagnosis to first documented recurrence or death from any cause, whichever occurred first; because death from any cause without documented recurrence was counted as an event, the endpoint corresponds to disease-free (rather than strict recurrence-free) survival. DFS data were available for 391 patients, with 170 events (105 recurrences and 65 deaths without documented recurrence). Because recurrence documentation in retrospective records is less robust than vital-status documentation, DFS was treated as a secondary supportive endpoint.

In univariate Cox regression for DFS, the cervical Sarcopenia Index showed a similar protective association as for OS (HR 0.71, 95% CI 0.59–0.86, *p* < 0.001). Thoracic SI was also associated with DFS (HR 0.75, *p* = 0.007); thoracic MFI showed an association with DFS (HR 1.24, *p* = 0.015). BMI was not associated with DFS (HR 0.96, *p* = 0.58), nor was the BOA single-vertebra C3 SI (HR 0.97, *p* = 0.74).

In a multivariate Cox model adjusted for HN-CCI and secondary malignancy, the cervical SI remained associated with DFS (HR 0.76, 95% CI 0.62–0.92, *p* = 0.005; n = 254, events = 118). Kaplan–Meier median-split analyses paralleled the OS findings (cervical SI, log-rank *p* = 0.019; thoracic SI, *p* = 0.043; thoracic MFI, *p* = 0.007; BMI, *p* = 0.74; C3 SI, *p* = 0.68).

## 4. Discussion

This study shows that automated 3D volumetric body composition analysis using the BOA algorithm yields prognostically informative biomarkers in patients with surgically treated HNSCC that, in this cohort, provide information beyond BMI. The principal finding is that the cervical Sarcopenia Index (Muscle/Bone ratio, derived from C1–C7 volumetric data) is associated with overall survival in a minimally adjusted model adjusting for comorbidity burden and secondary malignancy, and that this association remains after extending the adjustment to age, sex, tumor site, UICC stage, and treatment modality (Table 4). The hazard ratio remained near 0.66–0.76 across model specifications, supporting robustness. This is clinically relevant because most HNSCC patients undergo neck CT but not routine abdominal or thoracic imaging.

### 4.1. Volumetric Versus Single-Slice Body Composition Analysis

Our volumetric 3D segmentation approach contrasts with the established single-slice C3 or L3 method. In an exploratory within-cohort comparison, the volumetric C1–C7 BOA Sarcopenia Index was prognostic while the BOA-derived single-vertebra C3 metric was not. Importantly, the BOA C3 metric in this study is a single-vertebra volumetric measurement, not the conventional manually segmented 2D cross-sectional area used in most HNSCC sarcopenia studies, and the comparison should therefore be interpreted as a comparison within the BOA-derived feature space rather than a definitive refutation of conventional 2D C3-based assessment. The BOA C3 SI showed no meaningful discriminative ability (C-index 0.52) and, in the head-to-head model (Table 3, Model I), contributed no additional prognostic information after the inclusion of the volumetric C1–C7 metric. The correlation between the BOA C3 and volumetric C1–C7 metrics was near zero in our data, despite their overlapping anatomical scope. This suggests that, in the BOA-derived feature space, a single-vertebra C3 metric captures different information than the integrated volumetric assessment across C1–C7. Jung et al. [13] demonstrated in 36,317 UK Biobank participants that volumetric body composition measures had stronger mortality associations than single-slice metrics after multivariable adjustment, and Künnemann et al. [12] showed in a two-center lung cancer study (n = 4709) that the volumetric Sarcopenia Index outperformed conventional L3-based measurements. In contrast, Anyene et al. [14] found similar prognostic performance between single-slice L3 and multi-slice T12–sacrum metrics in colorectal cancer, and Jeong et al. [25] likewise reported broadly comparable performance for volumetric and single-slice metrics in a colorectal cancer cohort. Our in-sample C3 comparison suggests that, within the BOA-derived feature space, volumetric C1–C7 assessment captures prognostic information not reflected by a single-vertebra C3 BOA metric.

### 4.2. The Sarcopenia Index as a Frame-Normalized Metric

A practical finding is that raw cervical muscle volume alone was not associated with OS, whereas the Muscle/Bone ratio was. The denominator (bone volume) effectively controls for body frame size without requiring height, a practical advantage when stature is missing from records. This parallels the approach by Jungbauer et al. [26], who used SM/B and (SM + VAT)/B ratios from thoracic BOA in a recurrent/metastatic HNSCC immunotherapy cohort (n = 49) and found significant correlations with inflammatory indices (dNLR r = –0.47). In the lung cancer literature, Künnemann et al. [12] similarly demonstrated that SI = Muscle/Bone outperformed all other volumetric features, including conventional height-indexed metrics.

### 4.3. BMI Paradox and the Case for Body Composition Assessment

The non-prognostic nature of BMI in this cohort (multivariate *p* = 0.15, C-index 0.55), in contrast to the cervical SI association, is consistent with the “BMI paradox” reported in HNSCC. Hobday et al. [7] showed in a meta-analysis that overweight HNSCC patients (BMI 25–30) have lower mortality than normal-weight patients, a paradox explained by BMI’s inability to differentiate muscle from fat. Vangelov et al. [27] demonstrated that sarcopenic obesity—combining depleted muscle with obesity—is associated with a fourfold increased risk of critical weight loss during radiotherapy, further illustrating BMI’s limitations. Our data provide additional quantitative evidence: the moderate correlation between SMI and BMI confirms that these metrics capture overlapping but distinct biological information.

### 4.4. Muscle Quality: The Myosteatotic Fat Index

The thoracic MFI (IMAT/TAT) was associated with OS in the minimally adjusted supportive model (HR 1.35, *p* = 0.002) and remained associated after extended oncologic adjustment (HR 1.26, *p* = 0.013). Muscle quality (myosteatosis) and muscle quantity relative to skeletal frame (cervical SI) thus appear to provide complementary prognostic information. Roberti et al. [28] showed that IMAT at C3 predicted PFS in HNSCC independently of muscle area. In the broader oncology literature, intramuscular fat infiltration (myosteatosis) has been increasingly recognized as a prognostic marker, sometimes superior to muscle mass alone [12,28]. The MFI—normalizing IMAT to total adipose tissue—may offer a more stable and interpretable metric than absolute IMAT volume.

### 4.5. Cervical Body Composition: Clinical Implications

The identification of cervical C1–C7 volumetric body composition as an independent prognostic factor has possible clinical relevance, but external validation is required before clinical implementation. Most HNSCC patients undergo neck CT but not abdominal imaging. Olson et al. [15] established C3-derived sarcopenia thresholds predicting L3-based sarcopenia in HNSCC, and Barajas Ordonez et al. [19] confirmed the prognostic value of C3 muscle metrics in 904 patients. Our study extends this by demonstrating that a combined C1–C7 volumetric measurement—automatically segmented by BOA—is independently prognostic after controlling for multiple confounders. This eliminates the need for manual segmentation, selection of specific vertebral levels, or conversion formulas.

Our cohort was restricted to surgically treated patients by design, so the behavior of the Cerv SI under definitive radiotherapy, chemoradiation, or brachytherapy cannot be assessed from these data. Organ-preserving and highly conformal modalities are nonetheless an informative setting in which to test the marker. Interventional radiotherapy (brachytherapy) confines dose to the target and spares adjacent organs at risk, thereby limiting dysphagia and consequent weight loss [29]; because treatment-related dysphagia and weight loss are themselves mediators of sarcopenic decline, a baseline measure of physiologic reserve may carry greater actionable value where modality choice can modulate that toxicity. The cervical compartment is also left undisturbed in non-operated patients, so both baseline and serial Cerv SI remain measurable, addressing the constraint noted above for surgically treated patients. Prospective evaluation of the Cerv SI in definitive radiotherapy, chemoradiation, and brachytherapy cohorts is therefore a natural extension of this work. Because BOA operates on imaging already acquired for routine staging, it could ultimately function as a background imaging biomarker embedded in standard radiology workflows, adding prognostic information without additional acquisition time, cost, or patient burden.

### 4.6. Integration with Inflammatory Biomarkers

The relationship between body composition and systemic inflammation is increasingly recognized. Jungbauer et al. [26] demonstrated that BOA-derived SM/B ratios are inversely correlated with neutrophil-driven inflammation indices in patients with HNSCC undergoing immunotherapy. Cho et al. [30] showed that sarcopenia combined with elevated NLR carries a worse prognosis than either alone. In our cohort, preoperative leukocyte count and maximum postoperative CRP did not independently contribute to survival prediction, likely because total WBC count is a crude proxy for the neutrophil-specific inflammatory pathway. Future studies incorporating differential blood counts and SIRI calculations may reveal synergistic prognostic models that combine body composition with inflammation.

### 4.7. Body Composition Versus Clinical Functional Status Assessment

ASA classification, a strong univariate predictor (HR 1.55, *p* < 0.001), remained associated with OS when the cervical Sarcopenia Index was included (Model F, ASA HR 1.26, *p* = 0.046; Cerv SI HR 0.76, *p* = 0.011). With extended oncologic adjustment (Model X3), the cervical SI remained significant (HR 0.76, *p* = 0.018), and ASA was attenuated to borderline significance (HR 1.24, *p* = 0.076). Both ASA and the cervical SI thus appear to contribute partially overlapping but complementary information about patient vulnerability, while BMI fails to capture this dimension at all. This is consistent with findings by Marschner et al. [24], who compared ASA, ACE-27, and ECOG in 302 HNSCC patients receiving adjuvant (chemo)radiotherapy and found that ASA and ACE-27 outperformed ECOG-PS in multivariate survival analysis. A systematic review by Pai et al. [31] similarly identified ASA as the most frequently employed comorbidity index in head and neck surgery (represented in 70 of 116 studies), with the CCI and ACE-27 most consistently predicting mortality. Wu et al. [32] confirmed ASA grade III–IV as an independent risk factor for postoperative complications and reduced survival among patients undergoing HNC surgery. The practical implication is that automated volumetric body composition analysis may complement clinical functional status assessment by providing an objective, imaging-based measure of physiologic reserve derived from existing diagnostic CT scans, helping to reduce inter-rater variability and the documentation gaps inherent in retrospective performance status data [22,23].

### 4.8. Limitations

This study has several limitations. First, the retrospective single-center design limits generalizability and is compounded by non-random availability of imaging. As shown in Supplementary Table S1, BOA-evaluable imaging coverage was strongly associated with UICC stage (χ^2^ = 70.29, df = 12, *p* < 0.001), with higher-stage patients more frequently undergoing thoracic and/or abdominal imaging; regional comparisons should therefore be interpreted as exploratory. Second, detailed CT acquisition parameters were not uniformly retrievable across the 2010–2019 retrospective cohort, potentially introducing technical heterogeneity, and BOA segmentation accuracy was not formally validated against manual segmentation in this HNSCC cohort. However, BOA has been externally validated; cervical use in the presence of dental artifacts and variable scan coverage warrants further validation, and our local visual quality-control review mitigates but does not replace formal manual validation. Third, we lacked neutrophil differential counts for SIRI calculation and serum albumin data; ECOG performance status was incompletely documented, necessitating ASA as a functional-status surrogate; and smoking and alcohol exposure were not uniformly recorded. p16 testing was performed in only 34 of 391 patients, precluding adequate analysis of HPV-associated oropharyngeal disease. Fourth, the female subgroup (n = 35) was underpowered for sex-stratified analysis, despite evidence that sarcopenia’s prognostic impact may differ by sex [15,33]. Fifth, the multivariate body composition models had relatively small sample sizes for the thoracic (n = 221) and abdominal (n = 159) subgroups. Sixth, tumor staging was based on retrieved institutional records and could not be independently re-staged; and for 54 patients with documented ongoing follow-up but without a specific last-contact date, vital status was verified by chart review where possible, with patients confirmed alive administratively censored at the institutional database snapshot date (28 January 2025). Finally, we did not assess longitudinal changes in body composition; Mascarella et al. [34] recently demonstrated that sarcopenia trajectories predict survival in operable HNSCC, suggesting dynamic assessment may add prognostic value. A specific constraint applies to any such longitudinal use of the Cerv SI in this population. All measurements reported here were obtained from pretreatment staging CT acquired before surgery, so the present estimates are unaffected by operative tissue loss. Neck dissection, however, directly alters the cervical muscle and adipose compartments—particularly where the sternocleidomastoid or strap muscles are resected or devascularized, or the accessory nerve is sacrificed—and postoperative radiotherapy adds fibrosis and edema that change tissue volume and attenuation independently of muscle mass. Any postoperative change in the Cerv SI would therefore confound surgically induced tissue loss with systemic sarcopenic progression. Serial cervical assessment is likely to be interpretable only in non-operated patients, or against a postoperative rather than a pretreatment baseline; in surgically treated patients, a distant region such as the thoracic SI may be the more appropriate substrate for trajectory analysis.

## 5. Conclusions

In this retrospective single-center cohort of 391 surgically treated HNSCC patients, automated 3D volumetric body composition analysis using the BOA algorithm yielded composite indices that were independently associated with overall survival beyond BMI. The cervical C1–C7 Sarcopenia Index was the most robust marker, retaining independent significance after minimal adjustment for HN-CCI and secondary malignancy (HR 0.71, *p* = 0.002), after extended oncologic adjustment including UICC stage, age, sex, tumor site, and treatment modality (HR 0.72, *p* = 0.004), and in a sensitivity model additionally adjusting for inflammatory markers (HR 0.70, *p* = 0.008). These findings support exploration of automated volumetric body composition analysis as a complement to clinical risk assessment in surgically treated HNSCC and suggest that prognostic body composition information can be obtained from routine head and neck CT. Because discrimination remained modest and the study was retrospective and single-center, prospective multicenter validation, formal comparison with established C3 and L3 single-slice methods, and assessment of clinical decision impact (e.g., via decision-curve analysis) are warranted before routine clinical use Supplementary Materials.

## Figures and Tables

**Table 1:** Baseline characteristics of the study cohort.

Characteristic	Value	% or Range
Demographics		
Total patients	391	
Male/Female	356/35	91.0%/9.0%
Age at diagnosis (years)	60.9 ± 9.4	median 59.9
BMI (kg/m^2^)	26.6 ± 5.3	n = 377
HN-CCI	0.6 ± 0.8	n = 378
ASA classification		
ASA 1/2/3/4	7/210/154/7	1.9%/55.6%/40.7%/1.9%
Primary tumor site		
Larynx	151	38.6%
Oral cavity	140	35.8%
Oropharynx	61	15.6%
Hypopharynx	39	10.0%
Treatment modality		
Surgery alone	173	44.2%
Surgery + radiation	84	21.5%
Surgery + chemoradiation	134	34.3%
Biomarkers and comorbidity		
p16-positive (of tested)	17/34	50% of the tested
Secondary malignancy	67	17.1%
Outcomes		
Deaths	141	36.1%
Median follow-up (years)	5.0	IQR 2.6–6.3
BOA data availability		
Cervical (C1–C7)	256	65.5%
Thoracic	222	56.8%
Abdominal	159	40.7%
Body composition (mean ± SD)		
Cerv Muscle volume (mL)	1086 ± 487	n = 256
Cerv Sarcopenia Index	4.1 ± 1.0	
Thor Muscle volume (mL)	4635 ± 1148	n = 222
Thor Sarcopenia Index	2.0 ± 0.3	
Thor MFI (IMAT/TAT)	0.20 ± 0.05	
Abdominal muscle volume (mL)	6074 ± 1970	n = 159
Abd Sarcopenia Index	2.6 ± 0.4	
C3 Muscle volume (mL)	320 ± 116	n = 248
C3 Sarcopenia Index	3.7 ± 1.0	

Values are mean ± SD unless otherwise stated. BOA = Body and Organ Analysis; HN-CCI = Head and Neck Charlson Comorbidity Index; MFI = Myosteatotic Fat Index (IMAT/TAT).

**Table 2:** Univariate Cox proportional hazards analysis for overall survival. HRs are per 1-SD increase in standardized predictors.

Variable	n	Ev.	HR	95% CI	*p*	C-idx
Anthropometric						
BMI	377	134	0.838	0.70–1.00	0.052	0.547
Abdominal BOA						
Abd Muscle	159	68	1.008	0.79–1.28	0.951	0.492
**Abd SMI**	158	67	1.330	1.07–1.65	**0.009**	0.509
Abd SAT	159	68	0.909	0.70–1.18	0.468	0.557
Abd VAT	159	68	0.826	0.64–1.07	0.146	0.571
Abd IMAT	159	68	1.009	0.79–1.28	0.941	0.490
**Abd Sarc. Index**	159	68	0.799	0.62–1.02	0.077	0.553
Thoracic BOA						
Thor Muscle	222	86	0.855	0.67–1.10	0.218	0.563
**Thor SMI**	221	85	1.194	0.96–1.48	0.105	0.460
Thor SAT	222	86	0.794	0.63–1.00	0.051	0.564
Thor VAT	222	86	0.809	0.64–1.02	0.069	0.562
Thor IMAT	222	86	1.045	0.84–1.29	0.689	0.515
**Thor Sarc. Index**	222	86	**0.674**	0.53–0.86	**0.001**	0.602
**Thor MFI (IMAT/TAT)**	222	86	**1.382**	1.15–1.66	**<0.001**	0.621
Cervical BOA (C1–C7)						
Cerv Muscle	256	103	1.075	0.88–1.31	0.469	0.531
Cerv SMI	254	102	1.178	0.96–1.44	0.112	0.544
Cerv SAT	256	103	1.045	0.90–1.21	0.556	0.529
Cerv IMAT	256	103	1.073	0.89–1.30	0.465	0.520
**Cerv Sarc. Index**	256	103	0.662	0.54–0.81	**<0.001**	0.604
C3 Muscle (single-vertebra)	248	100	1.015	0.83–1.23	0.881	0.525
C3 Sarc. Index (single-vertebra)	248	100	1.013	0.83–1.24	0.899	0.520
Clinical confounders						
HN-CCI	378	134	1.189	1.01–1.40	**0.035**	
**ASA classification**	378	134	**1.550**	1.30–1.85	**<0.001**	
**Secondary malignancy**	391	141	**1.488**	1.30–1.70	**<0.001**	

HR = hazard ratio; CI = confidence interval; C-idx = Harrell’s concordance index; Ev. = events (deaths). Bold indicates *p* < 0.05. HR < 1 indicates the protective effect of higher values. ASA was tested as a continuous variable. The BOA-derived single-vertebra C3 Sarcopenia Index is included for direct comparison with the volumetric C1–C7 SI.

## Data Availability

The datasets analyzed during the current study are not publicly available because they contain identifying clinical and imaging data from a single-center patient cohort. Anonymized data supporting the conclusions of this article will be made available by the corresponding author on reasonable request, subject to local data-protection regulations.

## Supplementary Materials

The following supporting information can be downloaded at: https://www.mdpi.com/article/10.3390/cancers18152371/s1, Figure S1—Cohort flow and BOA-evaluable imaging availability by region; Figure S2—Representative cervical BOA segmentations from the visual quality-control review; Figure S3—Scaled Schoenfeld residuals for the cervical Sarcopenia Index (Cerv SI) in the primary clinically adjusted Cox model; Table S1—Distribution of UICC stage and BOA-evaluable imaging availability; Table S2—(a) Univariate Cox C-index with bootstrapped 95% confidence intervals for individual predictors of overall survival; (b) Pairwise C-index differences with bootstrapped 95% confidence intervals.; Table S3—Proportional hazards assessment for the primary clinically adjusted Cox model (Model X1).
