# Supplementary material for: BOA-Derived Volumetric CT Body Composition Provides Prognostic Information Beyond BMI in Surgically Treated Head and Neck Squamous Cell Carcinoma: A Retrospective Cohort Study
Source: Cancers (Basel). 2026 Jul 23;18(15):2371. doi: 10.3390/cancers18152371 (PMC13464902; doi:10.3390/cancers18152371)
Supplement: Supplementary file 1 [file cancers-18-02371-s001.zip › cancers-4433300-supplementary.pdf]

Supplementary Materials

BOA-Derived Volumetric CT Body Composition Provides Prognostic Information Beyond BMI in Surgically Treated Head and Neck Squamous Cell Carcinoma: A Retrospective Cohort Study

This document contains three supplementary figures and three supplementary tables referenced in the main manuscript:

- Figure S1: Cohort flow and BOA-evaluable imaging availability by region.
- Figure S2: Representative cervical BOA segmentations from the visual quality-control review.
- Figure S3: Scaled Schoenfeld residuals for the cervical Sarcopenia Index (Cerv SI) in the primary clinically adjusted Cox model.
- Table S1: Distribution of UICC stage and BOA-evaluable imaging availability.
- Table S2: (a) Univariate Cox C-index with bootstrapped 95% confidence intervals for individual predictors of overall survival; (b) Pairwise C-index differences with bootstrapped 95% confidence intervals.
- Table S3: Proportional hazards assessment for the primary clinically adjusted Cox model (Model X1).

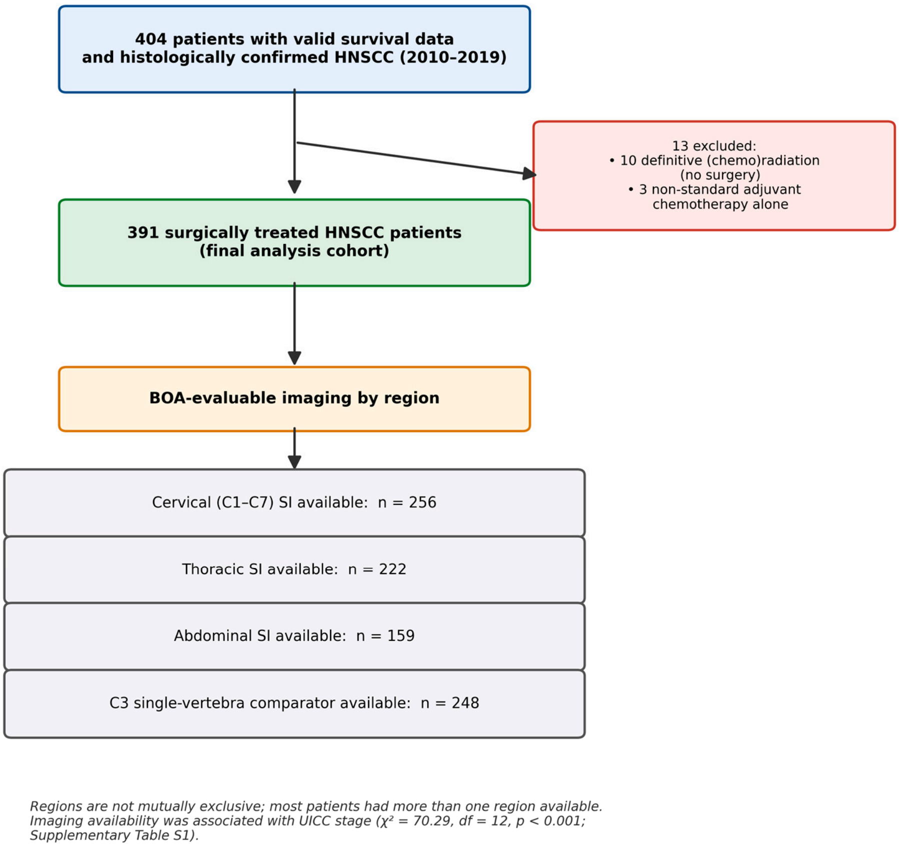

Figure S1. Cohort flow and BOA-evaluable imaging availability by region. Of 404 patients with valid survival data, 13 were excluded (10 definitive (chemo)radiation without surgery; 3 non-standard adjuvant chemotherapy alone), leaving 391 surgically treated HNSCC patients. Regions are not mutually exclusive. Imaging availability was associated with UICC stage ($\chi^2 = 70.29$, df = 12, $p < 0.001$; Table S1).

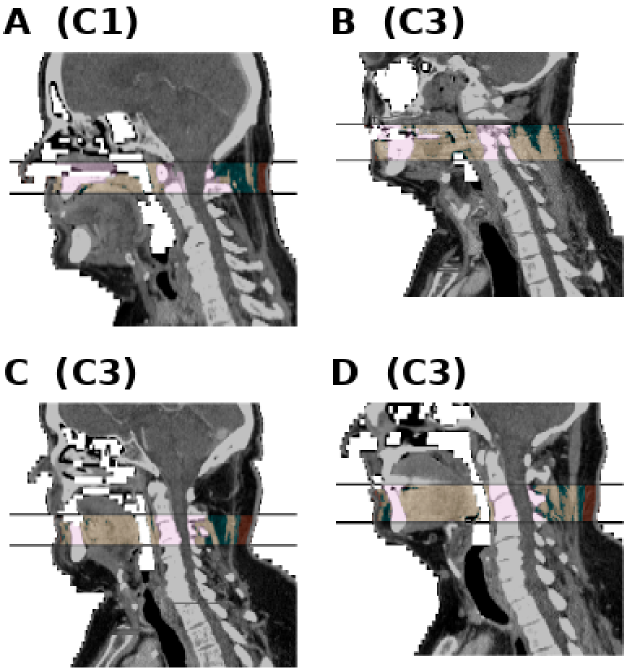

Figure S2: Representative cervical BOA segmentations from the visual quality-control review. Sagittal views with overlaid tissue segmentation at the C1 (A) and C3 (B–D) levels in four randomly selected cases; horizontal lines indicate the vertebral-level demarcation. No segmentation artifacts, vertebral-level mismatch, or incomplete C1–C7 coverage were identified in the reviewed subset.

Table S1. Distribution of UICC stage and BOA-evaluable imaging availability.

	No BOA-evaluable imaging	Cervical only	Cervical + thoracic	Cervical + thoracic + abdominal	Other combinations	Total
**UICC I**	37 (34.3%)	34 (31.5%)	15 (13.9%)	18 (16.7%)	4 (3.7%)	108 (100.0%)
**UICC II**	17 (23.3%)	20 (27.4%)	10 (13.7%)	20 (27.4%)	6 (8.2%)	73 (100.0%)
**UICC III**	11 (15.1%)	9 (12.3%)	18 (24.7%)	20 (27.4%)	15 (20.5%)	73 (100.0%)
**UICC IV**	20 (14.6%)	10 (7.3%)	22 (16.1%)	52 (38.0%)	33 (24.1%)	137 (100.0%)
**Total**	**85**	**73**	**65**	**110**	**58**	**391**

Pearson chi-squared test of independence: $\chi^2 = 70.29$, df = 12, $p < 0.001$. Imaging availability is strongly associated with UICC stage: higher-stage patients (III–IV) more frequently received multi-region imaging (thoracic and/or abdominal CT) than lower-stage patients (I–II). Among patients with no BOA-evaluable imaging, 36.5% were UICC III–IV; among those with cervical + thoracic + abdominal imaging, 65.5% were UICC III–IV. Row percentages are computed within each UICC stage. "Other combinations" include cervical + abdominal, thoracic-only, thoracic + abdominal, and abdominal-only imaging. The Sarcopenia Index (SI) status was determined by the per-region availability of all volumetric BOA outputs needed to compute the Muscle/Bone ratio for that region. This non-random distribution of imaging-region availability across UICC stage is the imaging-selection bias addressed in the main manuscript Limitations section.

Table S2a. Univariate Cox C-index with bootstrapped 95% confidence intervals for individual predictors of overall survival.

Predictor	n / events	HR per 1-SD	p-value	Harrell's C-index (95% CI)
**BMI**	377 / 134	0.84	0.052	0.547 (0.495–0.600)
**Cerv SI**	**256 / 103**	**0.66**	**<0.001**	**0.604 (0.541–0.666)**
**Thoracic SI**	**222 / 86**	**0.67**	**0.001**	**0.602 (0.537–0.669)**
**Thoracic MFI**	**222 / 86**	**1.38**	**<0.001**	**0.621 (0.566–0.685)**
**Abdominal SI**	159 / 68	0.80	0.077	0.553 (0.474–0.628)
**C3 SI (single-vertebra)**	248 / 100	1.01	0.899	0.520 (0.458–0.578)
**ASA**	**378 / 134**	**1.55**	**<0.001**	**0.599 (0.556–0.642)**
**HN-CCI**	378 / 134	1.19	0.035	0.545 (0.492–0.596)

Hazard ratios (HR) are per 1-SD increase in standardized predictor. Bootstrap 95% confidence intervals were derived from 1,000 resamples of patients with replacement. C-indices were computed from the univariate Cox linear predictor using Harrell's concordance index. A C-index of 0.50 indicates no discriminative ability over chance; bold rows are those whose lower bootstrap CI bound exceeded 0.50. Sample sizes vary because BOA-derived metrics required available regional imaging (see Table S1).

Table S2b. Pairwise C-index differences with bootstrapped 95% confidence intervals.

Comparison (A vs B)	n / events	C(A)	C(B)	Δ C (95% CI)	p-value
**Cerv SI vs BMI**	**253 / 101**	**0.602**	**0.516**	**+0.087 (+0.009 to +0.169)**	**0.038**
**Cerv SI vs C3 SI (single-vertebra)**	248 / 100	0.598	0.520	+0.078 (-0.010 to +0.169)	0.100
**Cerv SI vs Thoracic SI**	175 / 70	0.612	0.605	+0.007 (-0.080 to +0.094)	0.892
**Thoracic SI vs BMI**	220 / 84	0.607	0.542	+0.065 (-0.011 to +0.142)	0.092
**Thoracic MFI vs BMI**	**220 / 84**	**0.624**	**0.542**	**+0.082 (+0.014 to +0.144)**	**0.020**
**Cerv SI vs ASA**	254 / 102	0.604	0.567	+0.037 (-0.035 to +0.100)	0.264

Paired comparisons are restricted to patients with both predictors available (complete-case for each pair). For each comparison, both Cox models were fitted on the same patient subset using standardized predictors. Δ C = C(A) – C(B), with positive values indicating that predictor A discriminates better than predictor B. Bootstrap 95% confidence intervals were derived from 1,000 resamples of patients with replacement; two-sided bootstrap p-values were computed as twice the proportion of bootstrap Δ C values crossing zero in the unfavoured direction. Bold rows indicate p < 0.05. Key findings: the cervical Sarcopenia Index (Cerv SI) discriminates significantly better than BMI (Δ C = +0.087, p = 0.038), and the thoracic Myosteatotic Fat Index discriminates significantly better than BMI (Δ C = +0.082, p = 0.020). The cervical SI shows a non-significant numerical advantage over the single-vertebra C3 SI (Δ C = +0.078, p = 0.100). Importantly, when both regions are available, the cervical SI performs equivalently to the thoracic SI (Δ C = +0.007, p = 0.892), supporting the use of cervical CT alone for body composition assessment when thoracic imaging is unavailable; note, however, that this paired subset (n = 175) is enriched for higher UICC stage (64.0% UICC III–IV, versus 53.7% in the full cohort) because patients with multi-region imaging tend to have more advanced disease (see Table S1). Finally, the cervical SI does not significantly outperform ASA classification (Δ C = +0.037, p = 0.264), consistent with the observation in the main manuscript that the cervical SI and ASA capture overlapping aspects of patient physiologic reserve.

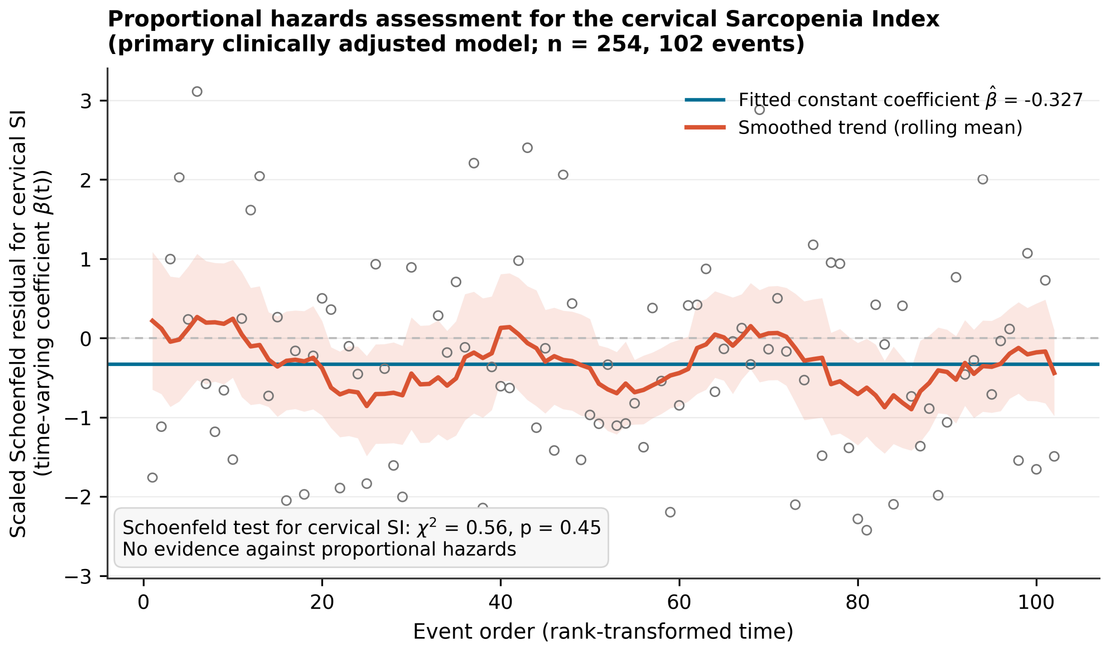

Figure S3. Scaled Schoenfeld residuals for the cervical Sarcopenia Index (Cerv SI) in the primary clinically adjusted Cox model (Model X1; n = 254, 102 events). Points show the scaled Schoenfeld residual for each event, centred on the fitted coefficient, plotted against rank-transformed time; the horizontal blue line is the fitted constant coefficient and the red line a smoothed trend with its 95% band. A time-varying coefficient would appear as systematic drift away from the horizontal line. No such trend is evident (Schoenfeld test χ^2 = 0.56, p = 0.45), supporting the proportional hazards assumption for the Cerv SI.

Table S3. Proportional hazards assessment for the primary clinically adjusted Cox model (Model X1). Scaled Schoenfeld residuals with rank-transformed time; n = 254, 102 events.

Covariate	χ^2	p-value
**Cervical Sarcopenia Index (Cerv SI)**	0.562	0.454
**Age**	1.094	0.296
**Sex (male)**	3.564	0.059
**HN-CCI**	1.310	0.252
**Secondary malignancy**	0.259	0.611
**UICC stage**	3.934	0.047
**Site: Oral cavity**	0.331	0.565
**Site: Oropharynx**	2.009	0.156
**Site: Hypopharynx**	4.751	0.029
**Treatment: Surgery + CRT**	2.953	0.086
**Treatment: Surgery + RT**	0.031	0.861
**GLOBAL test (df = 11)**	20.80	0.036

A significant p-value indicates evidence against the proportional hazards assumption for that covariate. The Cerv SI, the exposure of interest, satisfies the assumption (χ^2 = 0.562, p = 0.454). Minor departures are seen for sex (p = 0.059), UICC stage (p = 0.047), and the hypopharynx site indicator (p = 0.029), and the global test is correspondingly significant (χ^2 = 20.80, df = 11, p = 0.036). These departures involve adjustment covariates included to control confounding rather than to be interpreted individually, and none involves the Cerv SI; the primary estimate is therefore unaffected. The corresponding residual plot is shown in Figure S3.
